# Genetic and Phenotypic Analyses of a *Papaver somniferum* T-DNA Insertional Mutant with Altered Alkaloid Composition

**DOI:** 10.3390/ph5020133

**Published:** 2012-02-02

**Authors:** Noriaki Kawano, Fumiyuki Kiuchi, Nobuo Kawahara, Kayo Yoshimatsu

**Affiliations:** 1 Division of Tsukuba, Research Center for Medicinal Plant Resources, National Institute of Biomedical Innovation, 1-2 Hachimandai, Tsukuba, Ibaraki 305-0843, Japan; Email: kawahara@nibio.go.jp (N.K.); yoshimat@nibio.go.jp (K.Y.); 2 Faculty of Pharmacy, Keio University, 1-5-30 Shibakoen, Minato-ku, Tokyo 105-8512, Japan; Email: kiuchi-fm@pha.keio.ac.jp

**Keywords:** *Papaver somniferum* L., opium alkaloid, *Agrobacterium rhizogenes*, T-DNA insertional mutant

## Abstract

The *in vitro* shoot culture of a T-DNA insertional mutant of *Papaver somniferum* L. established by the infection of *Agrobacterium rhizogenes* MAFF03-01724 accumulated thebaine instead of morphine as a major opium alkaloid. To develop a non-narcotic opium poppy and to gain insight into its genetic background, we have transplanted this mutant to soil, and analyzed its alkaloid content along with the manner of inheritance of T-DNA insertion loci among its selfed progenies. In the transplanted T_0_ primary mutant, the opium (latex) was found to be rich in thebaine (16.3% of dried opium) by HPLC analysis. The analyses on T-DNA insertion loci by inverse PCR, adaptor-ligation PCR, and quantitative real-time PCR revealed that as many as 18 copies of T-DNAs were integrated into a poppy genome in a highly complicated manner. The number of copies of T-DNAs was decreased to seven in the selected T_3_ progenies, in which the average thebaine content was 2.4-fold that of the wild type plant. This may indicate that the high thebaine phenotype was increasingly stabilized as the number of T-DNA copies was decreased. In addition, by reverse transcription PCR analysis on selected morphine biosynthetic genes, the expression of codeine 6-*O*-demethylase was clearly shown to be diminished in the T_0_* in vitro* shoot culture, which can be considered as one of the key factors of altered alkaloid composition.

## 1. Introduction

Many attempts have been made to use breeding or molecular biological methods to modify the ability to produce secondary metabolites in medicinal plants. Among the challenges being addressed, manipulations of the morphine biosynthesis in the opium poppy (*Papaver somniferum* L.), particularly the conversion of narcotic morphine to codeine, which is of high importance as an antitussive and a synthetic source of dihydrocodeine, or to thebaine, which is also an important starting material for the semi-synthesis of the analgesic oxycodone, will contribute to the control of narcotics, and to the supply of useful alkaloids for the production of pharmaceuticals.

The gradual elucidation of enzymology of the alkaloid biosynthesis in *P. somniferum* led to genetical engineering of alkaloid biosynthetic pathway using native genes. The first report was on the introduction of a gene encoding berberine bridge enzyme (BBE) to *P. somniferum* in antisense orientation [1]. To date, several reports on metabolic engineering of *P. somniferum* have appeared, such as RNAi-mediated gene silencing of codeinone reductase (COR) [2], overexpression of COR [3], overexpression and antisense co-suppression of (*S*)-*N*-methylcoclaurine-3'-hydroxylase (CYP80B3) [4], overexpression and RNAi-mediated gene silencing of salutaridinol-7-*O*-acetyltransferase (SalAT) [5], and RNAi-mediated gene silencing of SalAT [6]. Mutant poppy *top1* [7] which accumulates thebaine and oripavine as major alkaloids instead of morphine was also established by the treatment of mutagen (ethyl methanesulphonate) and screening of progeny plants.

The T-DNA insertional mutant clone of *P. somniferum* PsM1-2, which we developed by the infection of the *Agrobacterium rhizogenes* strain MAFF03-01724, regenerated shoots from embryogenic callus that lacked the ability to produce morphine. Codeine was detected as a major alkaloid in this *in vitro* shoot culture [8]. By the improvement of the alkaloid analysis and proceeding studies on this mutant, thebaine (*ca.* 55 μg/g dry weight) and codeine (*ca.* 20 μg/g dry weight) were found to be the major opium alkaloids in the *in vitro* regenerated shoots [9]. The information provided from this mutant, which shows an altered alkaloid composition, might make an important contribution to the further modification of alkaloid production in *P. somniferum*, and therefore we carried out genetic and phenotypic analyses on this mutant.

Recently, long unidentified enzymes involved in the two demethylation steps in the conversion of thebaine to morphine were successfully identified as non-heme dioxygenases [10]. These two enzymes, namely, thebaine 6-*O*-demethylase (T6ODM) and codeine *O*-demethylase (CODM), represent the first known 2-oxoglutarate/Fe(II)-dependent dioxygenases that catalyze *O*-demethylation. The altered alkaloid composition in the PsM1-2 mutant may be due to the genetic mutation in the conversion steps from thebaine to morphine. In the present study, an expression analysis of these two enzymes together with selected genes involved in the morphine biosynthesis was carried out to reveal the molecular mechanism of the mutation.

## 2. Results and Discussion

### 2.1. Morphological Characteristics of the PsM1-2 Mutants

**Figure 1:**
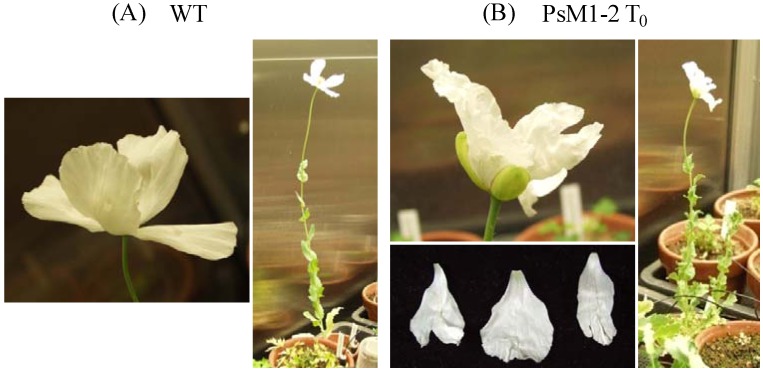
Appearances of the PsM1-2 T_0_ primary mutant and WT *P. somniferum* soil-cultivated in the phytotron. (**A**) WT, (**B**) PsM1-2 T_0_. Upper left: flower; right: grown plant; bottom left: petals with deep splits (PsM1-2 T_0_ only).

The days to flowering, number of petals, appearance of split on the boundary of the petal, and height of the aerial part at the seed-filling stage of soil-cultivated T_0_ mutant and selfed progenies are summarized in Table 1. The T_0_ primary mutant showed delay of flowering and dwarfness. In addition, a deep split was observed on the boundary of the petal (Figure 1). Delay of flowering was consistantly observed in the progenies. The number of petals, which was not altered at the T_0_, varied in the T_1_, T_2_ and T_3_ progenies. A deep split at the boundary of the petal was observed in 45% of T_1_ plants, 33% to 83% of T_2_ plants, and 8.3% and 10% of T_3_ plants.

**Table 1:** Summary of the morphological characteristics of PsM1-2 T_0_ mutant, selfed progenies, and WT plant.

Progenies	Lines	Number of Plants	Days to Flowering (Mean ± SD) (days)	Number of Petals: Percentage (%)	Split on Petal Boundary (%)	Plant Height (Aerial Part) (Mean ± SD) (cm)
T_0_	WT	1	47 *^1^	4: 100	0	60.0
	T_0_	1	71 *^1^	4: 100	100	38.0
T_1_	WT	6	53.5 ± 4.8	4: 100	0	42.4 ± 5.8
	T_1_	60	100.6 ± 14.6 ^####^	3: 1.7, 4: 41.7, 5: 35.0, 6: 16.7, 7: 3.3, 8: 1.7	45.0	52.1 ± 8.5 ^##^
T_2_	WT	12	53.3 ± 4.0	3: 25.0, 4: 75.0	8.3	36.0 ± 7.6
	#1-27(HT)	15	90.8 ± 12.6 ^####^	5: 60.0, 6: 33.3, 10: 6.7	60.0	44.7 ± 5.4 ^##^
	#2-17(HT)	6	79.8 ± 2.5 ^####^	5: 50.0, 6: 33.3, 8: 16.7	83.3	45.1 ± 3.4 ^#^
	#2-1(LT)	12	83.3 ± 6.8 ^####^	5: 66.7, 6: 16.7, 7: 8.3, 8: 8.3	33.3	35.6 ± 7.8
	#2-6(LT)	10	76.4 ± 3.6 ^####^	5: 10.0, 6: 40.0,7: 30.0, 8: 10.0,12: 10.0	80.0	39.4 ± 3.1
T_3_	WT	6	109.4 ± 0.9 *^2^	4: 100	0	80.3 ± 5.8
	#1-27(HT)L#2	10	129.2 ± 11.9 *^3, ###^	4: 40.0, 5: 50.0, 6: 10.0	10.0	45.8 ± 7.9 ^####^
	#2-17(HT)#2-1	12	131.1 ± 7.3 *^4, ####^	3: 8.3, 4: 75.0, 5: 16.7	8.3	47.0 ± 13.4 ^####^

*^1^: Days after transplanting; *^2^: n = 5; *^3^: n = 9; *^4^: n = 11; ^# ^*p* < 0.05; ^## ^*p* < 0.01; ^### ^*p* < 0.005; and ^#### ^*p* < 0.001 *vs.* WT.

### 2.2. Alkaloid Composition in the PsM1-2 Mutants

**Figure 2:**
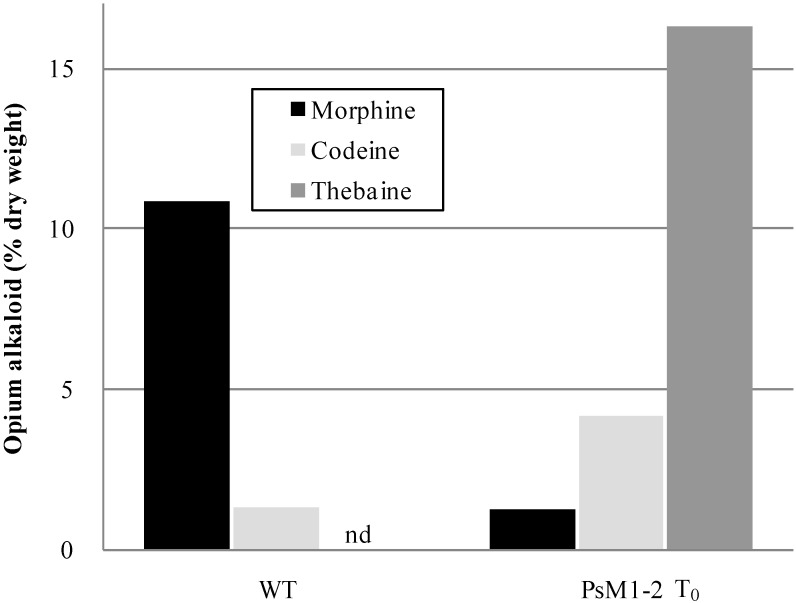
Alkaloid content in the latex from the soil-cultivated WT and PsM1-2 T_0_ mutant. nd: Not detected.

The soil-cultivated PsM1-2 T_0_ primary mutant accumulated 16.3% (% dry weight) of thebaine as a major opium alkaloid in the latex, which was not detected in the WT (Figure 2; Table 2). The morphine content in the mutant was 1.3%, which was *ca.* one tenth of that in the WT, and the codeine content was 4.2% in the mutant, *vs*. 1.3% in the WT.

**Figure 3:**
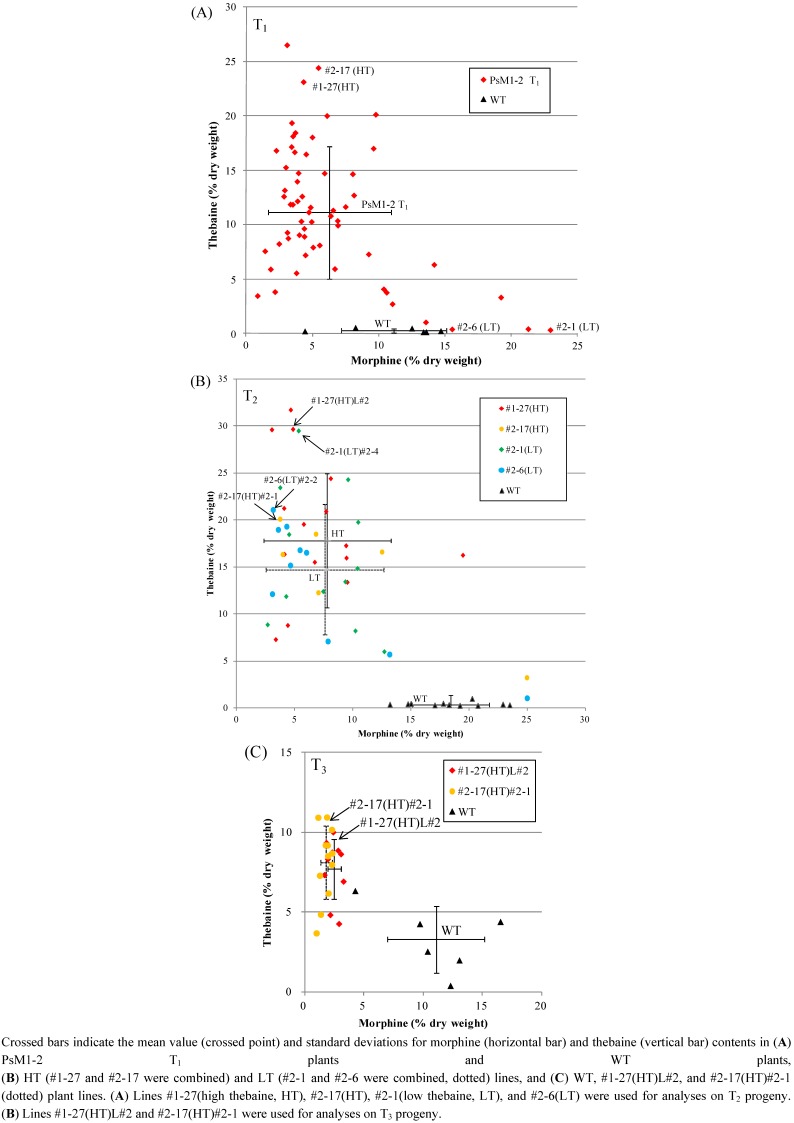
Alkaloid content in the latex from the soil-cultivated WT and PsM1-2 T_0_ mutant. nd: Not detected. Scatter diagram of the morphine (x-axis) and thebaine (y-axis) contents in (**A**) PsM1-2 T_1_ plants (n = 60) and WT plants (n = 6), (**B**) four lines of PsM1-2 T_2_ plants and WT plants, and (**C**) two lines of PsM1-2 T_3_ plants and WT plants.

The alkaloid compositions in the dried opium of selected progenies are summarized in Table 2, and the morphine and thebaine contents of the T_1_, T_2_ and T_3_ plants are plotted on a scatter diagram (Figure 3). The HPLC chromatograms of the representative lines of the T_1_ plants, WT plant, and authentic standards are shown in the Supplementary Figure 1.

**Table 2:** Opium alkaloid contents in PsM1-2 T_0_ mutant and selfed progenies.

Progenies	Lines	Number of plants	Morphine	Codeine	Thebaine	Papaverine	Noscapine
T_0_	WT	1	10.9	1.3	nd *	2.0	9.2
	T_0_	1	1.3	4.2	16.3	2.3	10.2
T_1_	WT	6	11.2 ± 4.0	2.6 ± 2.1	0.3 ± 0.2	2.4 ± 0.7	11.6 ± 4.3
	T_1_	60	6.3 ± 4.6 ^#^	3.8 ± 1.5	11.1 ± 6.1 ^###^	1.6 ± 0.5	7.9 ± 2.1 ^###^
Selected lines (T_1_)	#1-27(HT)	-	4.3	5.1	23.1	2.3	7.2
	#2-17(HT)	-	5.5	3.7	24.4	1.9	6.8
	#2-1(LT)	-	23.0	1.3	0.3	1.8	8.4
	#2-6(LT)	-	13.6	2.1	1.0	1.5	6.9
T_2_	WT	11	18.4 ± 3.3	1.5 ± 0.9	0.4 ± 0.2	2.7 ± 1.0	18.4 ± 4.3
	#1-27(HT)	15	7.0 ± 4.1 ^###^	5.8 ± 1.6 ^###^	19.1 ± 7.3 ^###^	2.2 ± 0.4	9.6 ± 2.4 ^###^
	#2-17(HT)	6	9.8 ± 8.1 ^#^	6.0 ± 0.5 ^###^	14.5 ± 6.1 ^###^	3.0 ± 0.4	9.2 ± 1.7 ^###^
	#2-1(LT)	12	7.6 ± 3.3 ^###^	5.8 ± 1.1 ^###^	15.9 ± 7.2 ^###^	2.8 ± 0.8	8.0 ± 2.0 ^###^
	#2-6(LT)	10	7.6 ± 6.8 ^###^	4.6 ± 1.3 ^###^	13.4 ± 6.7 ^###^	2.7 ± 0.5	11.7 ± 2.7 ^###^
Selectedlines (T_2_)	#1-27(HT)L#2	-	4.9	6.1	29.6	2.4	9.4
	#2-17(HT)#2-1	-	3.7	6.5	20.0	3.1	10.4
	#2-1(LT)#2-4	-	5.3	4.3	29.4	3.1	9.1
	#2-6(LT)#2-2	-	3.1	4.9	21.1	2.7	13.2
T_3_	WT	6	11.1 ± 4.1	1.5 ± 0.5	3.3 ± 2.1	1.9 ± 0.5	4.2 ± 1.6
	#1-27(HT)L#2	10	2.5 ± 0.6 ^###^	4.3 ± 0.4 ^###^	7.7 ± 1.9 ^##^	1.2 ± 0.1 ^###^	5.1 ± 0.7
	#2-17(HT)#2-1	12	1.8 ± 0.5 ^###^	2.9 ± 0.5 ^###^	8.1 ± 2.3 ^###^	1.1 ± 0.2 ^###^	4.1 ± 0.8

Mean value of the alkaloid content (% dry weight) with standard deviation (mean ± SD) for each line and the alkaloid content of selected lines are summarized. nd *: Not detected; ^# ^*p* < 0.05; ^## ^*p* < 0.005; and ^### ^*p* < 0.001 *vs.* WT.

The thebaine content in T_1_ plants varied widely, from 0.3% to 26.5%. From these plants, two high thebaine lines, #1-27(HT) (thebaine content: 23.1%) and #2-17(HT) (24.4%), and two low thebaine lines, #2-1(LT) (0.3%) and #2-6(LT) (1.0%), were selected and subjected to analysis of the T_2_ progeny. Interestingly, most of the progeny plants from both the HT and LT lines showed the high thebaine phenotype. From the T_2_ lines, two lines, #1-27(HT)L#2 (thebaine content: 29.6%) and #2-17(HT)#2-1 (20.0%), were selected for the analysis of T_3_ progeny.

**Figure 4:**
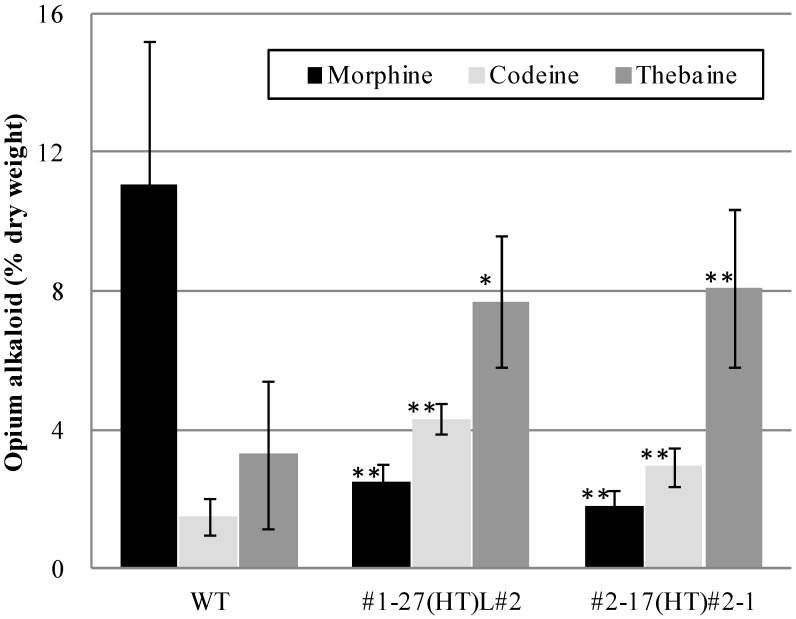
Morphine, codeine, and thebaine contents in T_3_ progeny. Mean value of six (WT), 10 [#1-27(HT)L#2], and 12 [#2-17(HT)#2-1] plants. Bars indicate standard deviation. * *p* < 0.005 and ** *p* < 0.001 *vs.* WT.

The thebaine content in T_3_ plants ranged from 4.2% to 10.0% in #1-27(HT)L#2 and from 3.7% to 10.9% in #2-17(HT)#2-1. The average thebaine content in T_3_ plants (two lines combined) was 2.4-fold of that in the WT; in contrast, the average morphine content decreased to *ca.* one fifth of that in the WT (Figure 4).

### 2.3. T-DNA Insertion Loci Analysis by IPCR and AL-PCR

**Figure 5:**
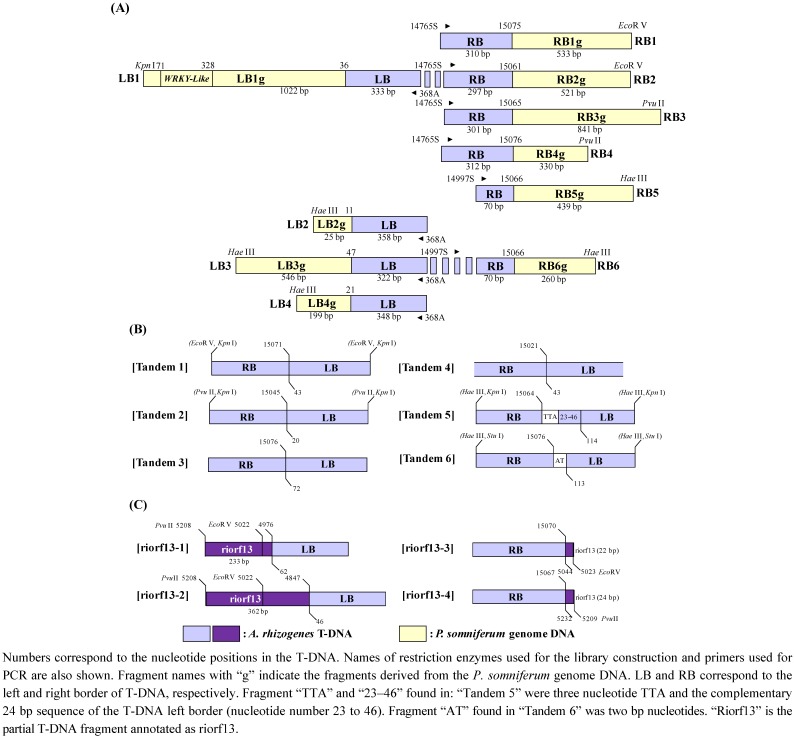
Schematic diagram of amplified fragments obtained in the analyses of T-DNA insertion loci in T_0_. (**A**) T-DNAs connected with *P.somniferum* genome (10 types, eight sites), (**B**) T-DNAs connected in tandem (six types), (**C**) T-DNAs connected with orf13 fragments (four types).

The genomic DNA regions adjacent to the inserted T-DNA borders were analyzed by the IPCR and AL-PCR methods. The obtained DNA fragments are summarized in Supplementary Table 1 and Figure 5 along with the PCR methods, the combination of template circular or adaptor-ligated genome DNA libraries, and the primer sets. Sequence analysis of the amplified products revealed that the fragments were classified into three types, (A) T-DNAs connected with *P. somniferum* genome DNA, (B) T-DNAs connected in tandem, and (C) T-DNAs connected with T-DNA internal fragments, as shown in Figure 5.

Type (A) includes four types of genome DNA fragments adjacent to T-DNA LB, and six types of genome DNA fragments adjacent to T-DNA RB. Of these fragments, LB1g and RB2g, LB3g and RB6g were confirmed to be both ends of single genomic loci, by PCR over the LB and RB genomic regions. Although the tally of paired border for other fragments were not found, at least eight independent T-DNA integrated sites, namely RB1, RB2 (LB1-RB2), RB3, RB4, RB5, LB2, LB3 (LB3–RB6), and LB4 were estimated to exist in the T_0_ plant.

A DNA fragment homologous (59% identity at the amino acid level) to the WRKY4 transcription factor (DDBJ/EMBL/GenBank accession no. AF425835) from *A. thaliana* was found in the LB1g region, at 695–952 bp 5' upstream of the junction. The DNA sequence of LB1g, which included a *WRKY*-like gene, was deposited in the DDBJ/EMBL/GenBank under accession No. AB574419.

No other gene with significant homology was found by the BLAST search tool in the genomic DNA regions adjacent to the inserted T-DNA.

Type (B) includes six types of DNA fragments. RB and LB were connected in a tail-to head manner at different junctions, or short DNA fragments were sandwiched. The fragment “Tandem 3” was found only in the genome direct amplification of T-DNA borders.

Type (C) consists of four DNA fragments. Two of the fragments were made up of short partial fragments of T-DNA riorf13 attached to an LB, and the other two were made up of RB attached to a fragment of T-DNA riorf13. In summary, T-DNA border fragments found were at eight independent sites of T-DNA integration, six borders of T-DNAs connected in tandem, and four borders connected with T-DNA internal fragments.

As for the copy numbers, types (A) and (B) corresponded to eight and six copies of T-DNAs, respectively. In the case of type (C), LB and RB connected with riorf13 were possibly borders of independent T-DNAs or borders of the same T-DNA. Therefore, the copy number can be estimated as two at minimum to four at maximum. Finally, the T-DNA copy number in the PsM1-2 T_0_ primary mutant could be estimated as 16 to 18.

### 2.4. Analysis of the Heredity Manner of T-DNA Inserted Loci

**Figure 6:**
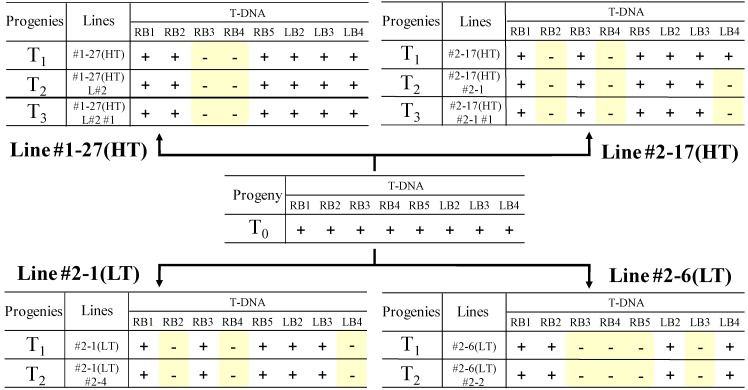
Inheritance of the eight independent T-DNA insertion loci (RB1, RB2, RB3, RB4, RB5, LB2, LB3, and LB4) in the representative four lines of selfed progenies. (+: Insertion locus detected; -: insertion locus not detected.)

The PCR analysis over T-DNA border and the adjacent genomic DNA found in the IPCR and AL-PCR analyses revealed that several T-DNA inserted loci were eliminated by selfing (Figure 6).

In the high thebaine line #1-27(HT), of the eight loci that were suggested to be independent T-DNA integration sites, RB3 and RB4 were eliminated in T_1_ progeny; in addition, in the high thebaine line #2-17(HT), RB2, RB4 were eliminated in T_1_ progeny and the additional elimination of LB4 was observed in T_2_ progeny. On the other hand, with respect to the LT lines that showed low thebaine content at T_1_ progeny, sites RB2, RB4, and LB4 were eliminated in #2-1(LT), and sites RB3, RB4, RB5, and LB3 were eliminated in #2-6(LT).

Notably thebaine content in these LT lines increased again in the T_2_ progeny to 29.4% in #2-1(LT)#2-4 and to 21.1% in #2-6(LT)#2-2 (Table 2) without a change in the T-DNA insertion pattern (Figure 6). These results imply that none of the eight T-DNA integrated loci were indispensable for the high thebaine phenotype.

### 2.5. T-DNA Copy Number Analysis by Real-Time PCR

**Figure 7:**
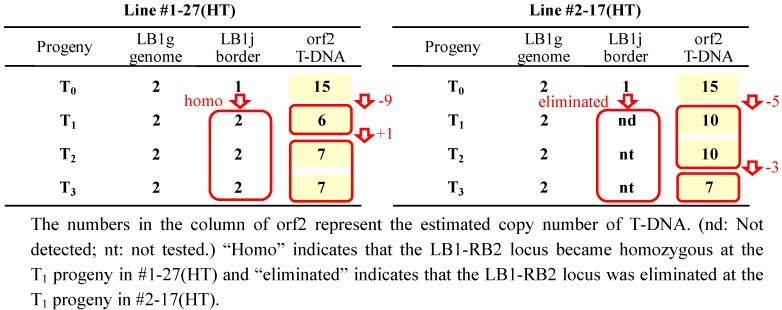
Shift of the relative abundance of target regions LB1g, LB1j, and orf2 of T-DNA insertion locus LB1-RB2 analyzed for two selfed lines, #1-27(HT) and #2-17(HT) by quantitative real-time PCR.

Standard curves for the quantification of the T-DNA copy number in T_0_ and selected progenies were prepared for each target region, LB1g, LB1j and orf2. The formulae and correlation coefficients were as follows: LB1g: y = −1.39ln(x) + 23.82 (r^2^ = 0.991); LB1j: y = −1.44ln(x) + 23.38 (r^2^ = 0.994); and orf2: y = −1.43ln(x) + 23.75 (r^2^ = 0.997). The relative abundances of each region in the samples were calculated by these formulae from the value of Delta Rn. The relative abundances in whole numbers, when the abundance of LB1g was set as 2, were LB1g:LB1j:orf2 = 2:1:15 in T_0_. And for T_1_[#1-27(HT)] and its progenies, the abundances were as follows (in the order of LB1g:LB1j:orf2): T_1_[#1-27(HT)], 2:2:6; T_2_[#1-27(HT)L#2], 2:2:7; and T_3_[#1-27(HT)L#2#1], 2:2:7. And for T_1_[#2-17(HT)] and its progenies, the values were as follows: T_1_[#2-17(HT)], 2:nd:10; T_2_[#2-17(HT)#2-1], 2:nt:10; and T_3_[#2-17(HT)#2-1#1], 2:nt:7 (nd: not detected; nt: not tested). These results are summarized in Figure 7.

For the abundance of LBj and orf2 in the #1-27(HT) series, the LB1-RB2 T-DNA insertion locus was estimated to become homozygous at the T_1_ progeny, as indicated by the doubled abundance of LB1j in T_1_. And the T-DNA copy number, estimated by the abundance of the orf2 region, was drastically decreased from 15 to six in T_1_, then increased to seven in T_2_ and kept at seven in T_3_. These data imply that more than half of the total T-DNA copies were eliminated in the first selfing. For the #2-17 series, the LB1j region was not detected in T_1_, which was consistent with the elimination of the LB1-RB2 T-DNA insertion loci in T_1_ revealed by the T-DNA insertion loci analysis (Figure 6). The abundance of orf2 was decreased from 15 to 10 in T_1_, and then decreased again from 10 to seven in T_3_, which implies that more than half of the T-DNA copies in the #2-17(HT) series were also eliminated by repeated selfing.

### 2.6. Expression Analyses on Morphine Biosynthetic Genes by RT-PCR

Firstly we tried to apply realtime-PCR for the expression analysis of morphine biosynthetic genes including *T6ODM*, *CODM* using the primer sequence reported by Hagel and Facchini [10]. However, prior to run the realtime-PCR, we found that PCR with these primers using our cDNA as a template gave multiple products. Although we have designed several primers, they could not make the PCR product as a single band. It may be attributed to the relatively high sequence homology of coding region among *T6ODM*, *CODM*, and *DIOX2*. Therefore we hired the semi-quantitative RT-PCR method for the expression analysis. To distinguish RT-PCR products between *T6ODM* and *CODM*, primers were designed to give different product size, *i.e.*, 549 bp for *T6ODM* and 411 bp for *CODM*.

**Figure 8:**
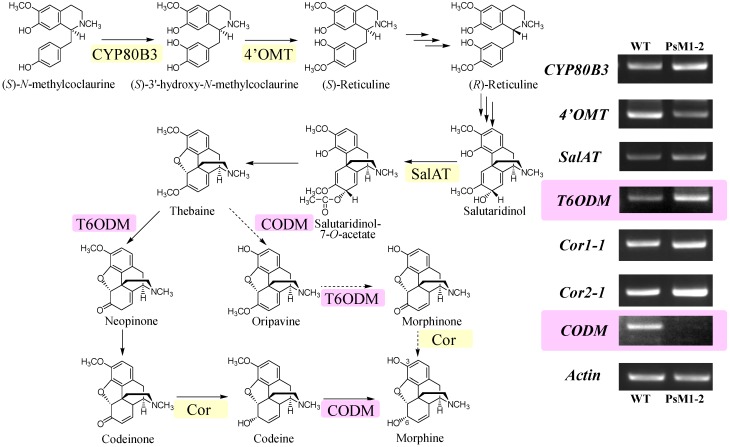
The morphine biosynthetic pathway downstream of (*S*)-*N*-methylcoclaurine with the results of expression analysis of selected morphine biosynthetic genes, *CYP80B3*, *4'OMT*, *SalAT*, *T6ODM*, *COR* (alleles *Cor1-1* and *Cor2-1*), and *CODM* by RT-PCR. Actin was used as an experimental control. Presumably, the pathway via oripavine (dotted pathway) does not exist in the *P. somniferum* Japanese cultivar “Ikkanshu” which we have used in this study [11,12].

Expression analysis on selected morphine biosynthetic genes downstream of (*S*)-*N*-methylcoclaurine revealed that the expression of *CODM* was completely diminished in PsM1-2 (Figure 8). On the other hand, the expression of *T6ODM* seemed to be slightly up-regulated in the PsM1-2 compared with the WT plant. Specific amplification of these two genes was confirmed by the comparison of the size of the bands and their calculated amplicon size. In PsM1-2, the expression levels of *CYP80B3* and *SalAT* seem to be slightly higher than WT, whereas *4'OMT* seems to be down-regulated. No significant difference in the expression level of genes was observed between PsM1-2 and WT for *Cor1-1* or *Cor2-1*.

### 2.7. Discussion

Morphological abnormalities, such as varied numbers of petals and splits on the boundary of petals, were frequently observed in the selfed progenies in the present study. However, no clear correlation was found between these morphological abnormalities and altered alkaloid compositions. Therefore, these findings were thought to be independent of the mutation in the secondary metabolism.

At the T_2_ generation, difference between high thebaine line and low thebaine line which was obvious at T_1_ generation, has disappeared. If the high thebaine phenotype is cause by the single mutation of the locus by T-DNA insertion, low thebaine phenotype should be dominant in the progeny plants. However, as observed in Figure 3, most of the progeny plants of low thebaine T_1_ lines have gained high thebaine phenotype again, which indicates that the multiple loci are responsible for the high thebaine phenotype. For the reason of this phenomenon, it is also possible that, methylation or suppression has occurred in unstable manner on the alkaloid biosynthesis related genes caused by the multiple T-DNA insertion events.

The content of thebaine, which was the major alkaloid in the latex of the mature plants of the mutants, varied widely in the T_1_ progeny. But by repeated selfing, in the T_3_ progeny, although the maximum content of thebaine (10.9%) was not particularly high, the range of thebaine content was much narrower than that in the T_1_ and T_2_ progenies. When the value of CV (coefficient of variation: standard deviation/average value) for the thebaine content was compared among T_1_, T_2_, and T_3_ progenies, it was 0.54 in T_1_, 0.40 in T_2_ (two HT lines combined), and 0.26 in T_3_ (two lines combined). And the CV for the morphine content was 0.74 in T_1_, 0.70 in T_2_ (two HT lines combined), and 0.28 in T_3_ (two lines combined). These lines of evidence indicate that the high thebaine (2.4-fold and 2.5-fold of WT in T_3_ #1-27(HT)L#2 and #2-17(HT)#2-1, respectively) and low morphine (0.2-fold of WT in both T_3_ #1-27(HT)L#2 and #2-17(HT)#2-1, respectively) phenotypes were stabilized by repeated selfing.

Analyses of the T-DNA integration sites and T-DNA copy number on the primary T_0_ mutant revealed that at least eight integration sites exist and as many as 18 copies of T-DNAs were estimated to be integrated into the genomic DNA in a highly complicated manner. Considering the complexity of the T-DNA integration, the IPCR, AL-PCR, and real-time PCR methods employed in this study can be considered as the most suitable methods for T-DNA insertional analysis, and more suitable than Southern blotting, whose signals may be beyond interpretation in this context. The number of T-DNA copies in PsM1-2 was too large for the transgenes integrated by genetical transformation. The presence of high numbers of transgenic insertions can lead to poor expression of transgenes through silencing. In this study, we tried to simplify the T-DNA integration structure and stabilize the high thebaine phenotype, and then to gain insight into the genetic factors for the altered alkaloid composition by obtaining selfed progenies. The T-DNA integration sites in PsM1-2 were paired to be homozygous or dropped off by selfing, and finally became half of the T_0_ in the selected T_3_ progenies. Although it is possible that other T-DNA copies were not detected, no correlation was found between any of the T-DNA integration sites and the altered alkaloid composition, by considering these data together, a reduction in the T-DNA copy number seems to have resulted in the stabilization of the high thebaine phenotype. Although it is hard to confirm, there is also a possibility that genome reorganization independent of T-DNA insertion has occurred during shoot regeneration or long term maintenance of *in vitro* culture. As we have accomplished the stabilization of high thebaine phenotype by selfing up to T_3_ generation, backcross experiment utilizing these selfed progeny plants is in progress.

In this study, the only gene homologous to the known gene found at the T-DNA integration loci was the *AtWRKY4* gene homologue found in the 5' upstream region of LB1g. As some type of WRKY transcription factor may function as a transcriptional regulator of benzylisoquinoline alkaloid biosynthesis in *Coptis japonica* Makino [13], the contribution of this locus to the altered alkaloid composition in the mutant was suspected. However, analysis of the T-DNA heredity manner indicated that the T-DNA insertion at LB1-RB2 region was not essential for the high thebaine phenotype.

The expression analyses on selected morphine biosynthetic genes, including two novel demethylases, *T6ODM* and *CODM*, between the *in vitro* shoot culture of the PsM1-2 mutant and seedlings of the WT plant revealed that the expression of *CODM* was fully suppressed in the mutant. Although the correlation between the transcript level of biosynthetic genes in young organs, such as seedling or *in vitro* shoot culture, and the alkaloid composition in the latex of mature plant needs to be clarified, the observed differences between the wild type plant and the mutant can be correlated to the alkaloid composition difference in them (morphine was detected in the WT, however, almost no morphine in the mutant [8]).

Kinetic studies on recombinant T6ODM and CODM from *P. somniferum* [10] have revealed that oripavine is the most preferred substrate of T6ODM, followed by thebaine, while codeine is not accepted as a substrate. On the other hand, CODM showed a higher preference for codeine than thebaine. Considering the substrate preference of these two demethylases, thebaine can be accumulated solely only under the condition that the expression of both *T6ODM* and *CODM* is suppressed, and the suppression of *CODM* may result in accumulation of codeine. In actuality, however, a large amount of thebaine with a smaller amount of codeine is accumulated in the latex from mature plants of PsM1-2 mutants. This pattern of compounds detected in the mutant is similar to that of the *T6ODM*-silenced transformant by virus-induced gene silencing [10]. In contrast, the *CODM*-silenced transformant accumulates mainly codeine, together with smaller amounts of thebaine and morphine [10]. Although the alkaloid productivities of those transformants cannot be simply compared with PsM1-2, as the alkaloid composition varies highly even among the cultivars [14], it is assumed that suppression of CODM did not simply lead to the thebaine accumulation in PsM1-2. And it is also possible that in a Japanese cultivar that does not have the pathway from thebaine to morphine via oripavine [11,12], the substrate preferences of T6ODM and CODM differ from those of oripavine-producing cultivars.

As the regulation of opium alkaloid production in *P. somniferum* is highly complicated and varies among cultivars—and even among the developmental stages [15,16] or individual parts of a single plant [17]—further detailed studies on the molecular regulation of alkaloid production, such as expression analyses of *T6ODM* and *CODM* in the latex-producing capsule of PsM1-2, are required.

## 3. Experimental Section

### 3.1. Plant Materials

The wild type (WT) plant of *P. somniferum* L. used was the Japanese cultivar “Ikkanshu”, and the *A. rhizogenes* strain MAFF03-01724 T-DNA insertion mutant line was PsM1-2 [8]. The *in vitro* culture of PsM1-2 used in this experiment was previously subjected to a single round of cryopreservation and regenerated to plantlet on Murashige-Skoog (MS) solid media [18] by the method described previously [19,20] with slight modifications.

### 3.2. Maintenance and Cultivation of Plant Materials

The WT plant seeds were obtained from the field-grown plants at the Research Center for Medicinal Plant Resources, Division of Tsukuba.

The PsM1-2 T_0_* in vitro* shoot culture was maintained on MS solid media at 20 °C under a 14 h light/10 h dark condition and then transplanted in soil in a 9 cm diameter pot and acclimatized in a phytotron in 60% relative humidity under a cycle of 16 h light at 20 °C and 8 h dark at 17 °C.

Seeds of T_1_ plant obtained from the soil-cultivated plant of the PsM1-2 T_0_ primary mutant were sown on the soil in a 15 cm diameter pot and cultivated in a greenhouse under a 16 h light/8 h dark cycle at 20 °C and 60% relative humidity. Plants were fertilized with 500-fold diluted Hyponex^®^ (Hyponex Japan, Osaka, Japan) once a week.

T_2_ seeds from the two lines of T_1_ plants that showed high thebaine content and had abundant mature seeds were selected for cultivation of T_2_ progeny. The cultivation conditions were the same as for T_1_ plants.

T_3_ seeds from two lines of T_2_ plants with high thebaine content were germinated on rock wool with fertilization with 2,000-fold diluted Hyponex^®^ in a greenhouse under a 16 h light/8 h dark cycle at 20 °C and 60% relative humidity. After one month, seedlings were transplanted onto the soil in a 9 cm diameter pot, and grown in the growth chamber under a 12 h light/12 h dark condition (short day condition) at 20 °C and 60% relative humidity. *Ca.* 80 days after sowing, the lighting was changed to a long day condition of 16 h light/8 h dark at 20 °C and 60% humidity for flowering. After transplanting, plants were fertilized with 500-fold diluted Hyponex^®^ once a week.

For each experiment, WT plants were grown together as an experimental control. All self-pollination events were performed manually.

### 3.3. Phenotypic Observation of the PsM1-2 Mutants

Phenotypic parameters such as days to flowering, number of petals, appearance of splitting on the boundary of the petal, and the height of the aerial part at the seed-filling stage, were observed on each plant.

### 3.4. HPLC Analysis of Alkaloid Content in the Latex

The opium alkaloid content in the latex was analyzed by HPLC. Latex was collected from the capsule of either the WT or mutant *P. somniferum ca.* two weeks after flowering, by incising the capsule surface. Collected latex was dried at 50 °C. Approximately 5 mg of dried latex was measured accurately and subjected to alkaloid extraction by adding 5 mL of methanol followed by 30 min of sonication and mixing thoroughly using a tube mixer. After centrifugation at 20,000× *g* for 1 min, supernatant was applied to an Ultrafree-MC spin column (Millipore, Bedford, MA, USA) and centrifuged at 20,000× *g* for 1 min, and then 5 μL of the flow through was injected into an HPLC column. The HPLC conditions were as follows. HPLC instruments: Waters Alliance PDA System (separation module: 2795; photodiode array detector: 2996) (Waters, Milford, MA, USA). Column: TSK-GEL ODS100V (pore size 5 μm, φ4.6×250 mm) (Tosoh, Tokyo, Japan). Solvent system: CH_3_CN (A), 10 mM sodium 1-heptanesulphonate (pH 3.5) (B). Solvent gradient (A%): 0 min 28%, 15 min 34%, 25 to 39 min 40%, 40 min 28%. Detection: UV 200 to 400 nm (spectrometric identification of compounds), UV 284 nm (quantitative analysis). Column temperature: 30 °C. Flow rate: 0.7 mL/min. HPLC data were collected and analyzed by an Empower system (Waters).

Alkaloid components were identified by the comparison of retention time and the UV spectra with authentic standards. Morphine hydrochloride and codeine phosphate were purchased from Takeda Pharmaceutical Company Limited (Osaka, Japan). Oripavine was a gift from Einar Brochmann-Hanssen (University of California, San Francisco, CA, USA). Magnoflorine iodide and jateorrhizine were gifts from Akira Ikuta (Science University of Tokyo, Japan). Reticuline and columbamine were gifts from Fumihiko Sato (Kyoto University, Japan). Isothebaine was isolated from *Papaver pseudo-oriental*e (Fedde) Medw. by our group. Thebaine was a gift from Ruri Kikura-Hanajiri (National Institute of Health Sciences, Japan). Papaverine hydrochloride, noscapine hydrochloride, coptisine chloride, sanguinarine chloride, and berberine chloride were purchased from Wako Pure Chemical Industries (Osaka, Japan). Alkaloid contents were calculated as a weight percent of the dried latex (opium).

### 3.5. Genomic DNA Preparation from P. somniferum

Genomic DNA was prepared from *ca.* 100 μg of fresh leaves of selfed plants grown in the growth chamber, or from *ca.* 100 μg of whole *in vitro* plantlet of the PsM1-2 T_0_ mutant, which mainly consisted of leaves and stems, by using a DNeasy Plant Mini Kit (Qiagen, Hilden, Germany) according to the manufacturer’s instructions.

### 3.6. Analysis of T-DNA Insertion Loci by IPCR and AL-PCR

The inverse-PCR (IPCR) method [21,22] and adaptor ligation PCR (AL-PCR) method were used for the analysis of the flanking unknown genome DNA sequence, adjacent to the inserted T-DNA. In this study, the Vectorette PCR method [23,24,25], an improved method of AL-PCR, was employed to reduce non-specific amplicons.

The genomic DNA library for each PCR method was constructed by digestion of genomic DNA by the appropriate restriction enzymes and self-ligation to form a circular DNA library, or an adaptor linker attached genome DNA library.

### 3.7. Genomic Library Construction for IPCR

Genomic DNA was digested with the restriction enzymes *Bam*HI, *Eco*RV, *Hae*III, *Kpn*I, *Pvu*II, *Ssp*I, or *Stu*I. Completely digested DNA was ligated by using a Fastlink^®^ DNA Ligation Kit (AR Brown, Tokyo, Japan) to form a circular genome DNA library.

### 3.8. Genomic Library Construction for AL-PCR

The sequences of the adaptor oligo DNA and adaptor specific primers used in this study are listed in Supplementary Table 2. Two complementary oligo DNAs, AP-LS and AP-SS, were annealed to form an adaptor unit. Genomic DNA was digested with the restriction enzymes *Eco*RV, *Hae*III, *Pvu*II, *Ssp*I, or *Stu*I, which produce blunted ends. The completely digested DNA was ligated with adaptor units by using a Fastlink^®^ DNA Ligation Kit to form an adaptor ligated genome DNA library.

### 3.9. IPCR and AL-PCR

Amplification of the target region was performed by the nested PCR method using TaKaRa Ex Taq^TM^ DNA polymerase (Takara Bio, Shiga, Japan) under the following conditions. The combinations of PCR methods, template genome DNA libraries and primer sets are listed in Supplementary Table 1. T-DNA-specific primers were designed based on the DNA sequence of the T-DNA region of the *A. rhizogenes* plasmid pRi1724 (DDBJ/EMBL/GenBank accession no. AP002086). The first PCR conditions were as follows: primary denaturation at 94 °C for 5 min; followed by 30 cycles of 94 °C for 1 min, 42 °C for 2 min, and 72 °C for 3 min; with a final extension at 72 °C for 10 min. After PCR, the solution was held at 4 °C. The first PCR reaction solution was applied to a SUPREC^TM^-02 filter (Takara Bio) to eliminate the primers and then used as a template for the second PCR. Second PCR conditions were as follows: primary denaturation at 94 °C for 5 min; followed by 30 cycles of 94 °C for 1 min, 48 °C for 2 min, and 72 °C for 3 min; with a final extension at 72 °C for 10 min. After PCR, the solution was held at 4 °C. The product of the second PCR was gel purified and cloned into the sequencing vector pT7-Blue^®^ (Novagen, Madison, WI). Propagated plasmid DNA was subjected to DNA sequencing using a BigDye^®^ Terminator v3.1 Cycle Sequencing Kit and ABI PRISM^®^ 3100—Avant Genetic Analyzer (Applied Biosystems Japan, Tokyo, Japan). Homology search was performed on T-DNA flanking genome DNA sequences with the BLAST tool at NCBI.

### 3.10. Direct Amplification of T-DNA Borders Connected in Tandem

PCR was performed on uncut genome DNA of T_0_ to amplify the border region of T-DNAs connected in tandem. The primers used are listed in Supplementary Table 1. The PCR conditions were the same as for the IPCR.

### 3.11. Analyses of T-DNA Insertion Loci and Heredity Manner by PCR

The PCR method was employed to confirm the T-DNA integration loci on *P. somniferum* genome DNA and to analyze the heredity manner in the selfed progenies.

To find out the tally of the paired genomic regions found adjacent to the T-DNA left borders (LBs) and right borders (RBs) revealed by IPCR and AL-PCR analyses, PCR amplification was performed with the pair of genomic region-specific LB and RB (e.g*.*, LB1g *vs*. RB2g) primers listed in Supplementary Table 3, under the following PCR conditions: primary denaturation at 94 °C for 5 min; followed by 30 cycles of 94 °C for 30 s, 58 °C for 30 s, and 72 °C for 1 min; with a final extension at 72 °C for 10 min. After PCR, the solution was held at 4 °C. TaKaRa Ex Taq^TM^ was used as the PCR polymerase. The PCR product was separated on agarose gel. The paired genomic regions, which gave a PCR product was judged as the single T-DNA integrated locus.

To judge whether or not the T-DNA integration loci were present in the selfed progenies, PCR amplification was performed between the genome region-specific primers listed in Supplementary Table 3 and T-DNA LB- or RB-specific primers (MAFF-226A or MAFF-14963S). PCR was performed under the following PCR conditions: primary denaturation at 94 °C for 5 min; followed by 30 cycles of 94 °C for 30 s, 58 °C for 30 s, and 72 °C for 1 min; with a final extension at 72 °C for 10 min. After PCR, the solution was held at 4 °C. GoTaq^®^ Green Master Mix (Promega, Madison, WI, USA) was used as the PCR polymerase. The PCR product was separated on agarose gel.

### 3.12. T-DNA Copy Number Analysis by Real-time PCR

**Figure 9:**
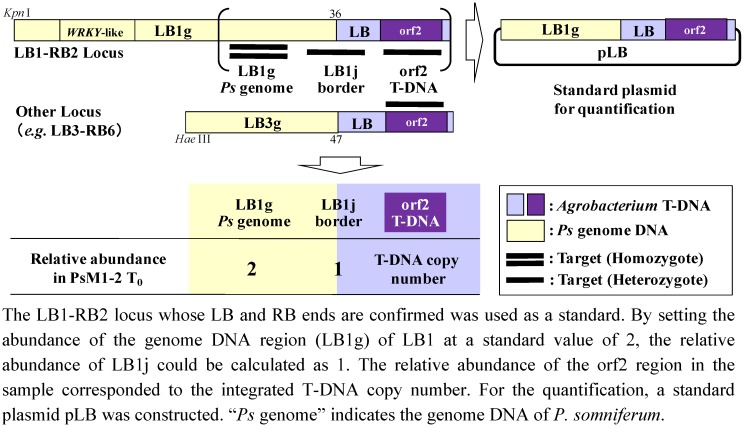
Schematic diagram of the strategy of T-DNA copy number analysis by real-time PCR.

The T-DNA copy number was analyzed by the quantitative real-time PCR method [26,27]. The strategy used for estimating the T-DNA copy number is as follows. When information of one of the integrated T-DNA sites was provided by T-DNA integrated loci analysis, and also, when the T-DNA integrated site was a single copy, the copy number of the integrated T-DNA could be calculated as a multiple of the relative abundance of standard DNA fragments, as shown in Figure 9.

This estimation method can be enacted under the hypotheses that (1) the genome DNA of PsM1-2 is diploid (2n = 22) [28], (2) all of the T-DNA is integrated into the host genome DNA in a heterozygous manner, and (3) one of the integrated T-DNAs for which both the LB and RB borders are known (e.g*.*, the LB1-RB2 locus) is a single copy.

Under these hypotheses, by comparing the relative abundance of the T-DNA internal region (in this case, orf2), the T-DNA—*P. somniferum* genome junction region (LB1j), and the *P. somniferum* genome region (LB1g), we can calculate the inserted T-DNA copy number by fixing the abundance of LB1g as two.

In our experiment, one of the T-DNA-integrated sites, LB1-RB2, which will be described in the Results section, was set as a standard. And for the quantification standard plasmid DNA, we constructed pLB1, which included, the LB1g, LB1j, and orf2 regions of the T-DNA. A DNA fragment with these three regions was amplified by PCR with the primers LB1-orf2-S (5'-CTC ATA AGC AGT GGT ATT GCT C-3') and LB1-orf2-A (5'-CGC ATT CAT GCG GTT ATG GAG-3') and KOD-Plus-DNA polymerase (Toyobo, Osaka, Japan) under the following PCR conditions: primary denaturation of 94 °C for 2 min; followed by 35 cycles of 94 °C for 15 s, 62 °C for 30 s, 68 °C for 90 s. After PCR, the solution was held at 4 °C. The amplified product was cloned into the pT7-Blue^®^ vector (Novagen) and then propagated in *E. coli*. The quantitative standard plasmid DNA pLB1 and genome DNA prepared from the primary T_0_ mutant and selected T_1_, T_2_, and T_3_ progenies of the PsM1-2 mutant were diluted serially with the dilution buffer supplied with the real-time PCR reagent SYBR^®^ Premix Ex Taq^TM^ II (Perfect Real Time; Takara Bio). Real-time PCR was run using the target region-specific primers listed in Supplementary Table 4 with the real-time PCR reagent on an ABI PRISM 7000 Sequence Detection System (Applied Biosystems Japan). The obtained data were analyzed using the supplied software (Applied Biosystems Japan) and the relative abundance of each target region was deduced from each Delta Rn value using standard curves. Standard curves for each target region were plotted with the plasmid concentration (fg/μL) on the x-axis and the Delta Rn on the y-axis. The curves showed good correlations. The relative abundances of each target region were calculated so that the abundance of the LB1g region was 2, and then rounded off to a whole number.

### 3.13. Actin Gene Amplification from P. somniferum

A fragment of actin cDNA was amplified by degenerate PCR using the forward primer 5'-AAR GCN AAY MGN GAR AAR ATG AC, and the reverse primer 5'-CCR TAN ARR TCY TTN CKD ATR TC, which were designed from the completely conserved regions of the amino acid sequences of other actins, such as *Arabidopsis thaliana* (*actin-1*: DDBJ/EMBL/GenBank accession No. M20016), *Nicotiana tabacum* (*actin*: X63603), and *Zea mays* (*Maz56*: U60514). cDNA synthesized from the total RNA of young seedlings of *P. somniferum* was used as a template for PCR. The manual hot-start procedure was used for the amplification. TaKaRa Ex Taq^TM^ DNA polymerase was added after primary denaturation at 94 °C for 5 min, and then the following protocol was carried out in a GeneAmp2400 thermal cycler (Applied Biosystems Japan): 30 cycles of 94 °C for 1 min, 48 °C for 2 min, and 72 °C for 3 min; with a final extension at 72 °C for 10 min. After PCR, the solution was held at 4 °C. The amplified fragment was cloned into the pT7-Blue^®^ vector followed by DNA sequencing. Two representative actin cDNA sequences, whose deduced amino acid sequences showed 92% and 95% identity to the *Arabidopsis actin-1*, were named *PsACT1* (AB574417) and *PsACT2* (AB574418), respectively.

### 3.14. Expression Analysis of the Morphine Biosynthetic Genes

The expression levels of selected morphine biosynthetic genes downstream of (*S*)-*N*-methylcoclaurine, *CYP80B3* (DDBJ/EMBL/GenBank accession no. AF134590 [29]), (*R*,*S*)-3'-hydroxy-*N*-methylcoclaurine 4'-*O*-methyltransferase (*4'OMT*; AY217333 [15]), *SalAT* (AF339913 [30]), *T6ODM* (GQ500139 [10]), *COR* (allele *Cor1-1*: AF108432; allele *Cor2-1*: AF108438 [31]), and *CODM* (GQ500141 [10]) in the WT plant and the PsM1-2 mutant were analyzed and compared by the reverse transcription PCR (RT-PCR) method.

Total RNA was prepared from the whole plants of two-week-old seedlings of field-grown WT *P. somniferum*, or from whole *in vitro* plantlet of the PsM1-2 T_0_ mutant, which mainly consisted of leaves and stems, by using an RNeasy Plant Mini Kit (Qiagen) according to the manufacturer’s instructions. One microgram of total RNA samples was subjected to single-stranded cDNA synthesis by reverse-transcription with oligo-(dT) primer (RACE32: 5'-GAC TCG AGT CGA CAT CGA TTT TTT TTT TTT TT-3') [32] using Superscript® II Reverse Transcriptase (Life Technologies, Carlsbad, CA, USA) according to the manufacturer’s instructions. Synthesized ss-cDNA was used as a template for PCR with the gene-specific primers listed in Supplementary Table 5. The PCR conditions were as follows: primary denaturation 94 °C for 5 min; followed by 30 cycles of 94 °C for 30 s, 58 °C for 30 s, and 72 °C for 1 min; with a final extension at 72 °C for 10 min. After PCR, the solution was held at 4 °C. PCR products were separated on 1.0% agarose gel and signal intensities were observed. The actin gene *PsACT1* from *P. somniferum* was used as an experimental control.

### 3.15. Statistical Analysis

Values were expressed as the mean ± standard deviations (SD) and were analyzed by the Tukey-Kramer multiple comparison test using the statistical analysis system “R” software package [33]; a *p* value of less than 0.05 was considered significant.

## 4. Conclusions

By combining genetic and phenotypic analyses of the T-DNA insertional mutant PsM1-2 with selfing, we have succeeded in stabilizing the high thebaine phenotype in coordination with a reduction in the number of inserted T-DNA copies. Although the genetic mode of CODM suppression in *in vitro* plantlet and of the accumulation of thebaine still remain unknown, studies on this mutant and its progenies may provide new insights into the molecular basis of morphine biosynthesis, and could ultimately allow us to manipulate the biosynthesis of this compound at will.

## Supplementary Files

Supplementary File 1: PDF-Document (PDF, 200 KB)
