# Supplementary material for: Genetic and Phenotypic Analyses of a Papaver somniferum T-DNA Insertional Mutant with Altered Alkaloid Composition
Source: Pharmaceuticals (Basel). 2012 Feb 2;5(2):133–54. doi: 10.3390/ph5020133 (PMC3763633; doi:10.3390/ph5020133)
Supplement: Supplementary File 1: — PDF-Document (PDF, 200 KB) [file pharmaceuticals-05-00133-s001.pdf]

Supplementary Figure 1. HPLC chromatograms of the authentic standards (**A**) and the methanol extracts from the dried latex of *P. somniferum* WT plant (**B**) and the representative transformants (**C** to **F**).

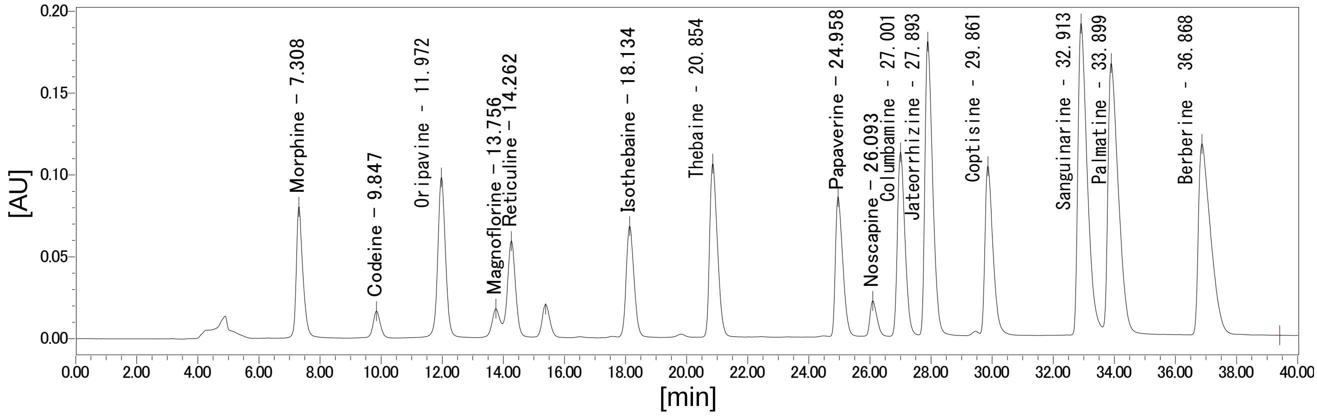

(**A**) Standards

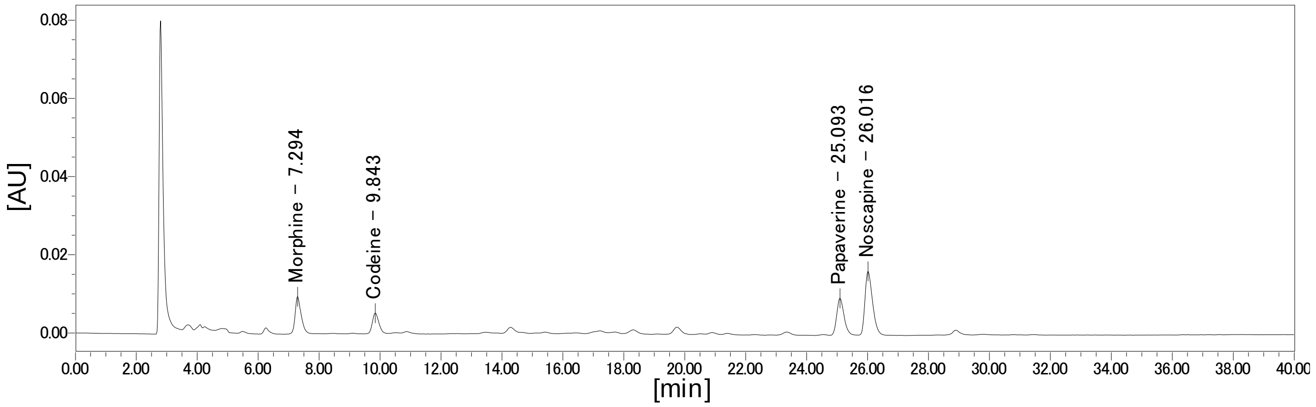

(**B**) WT1

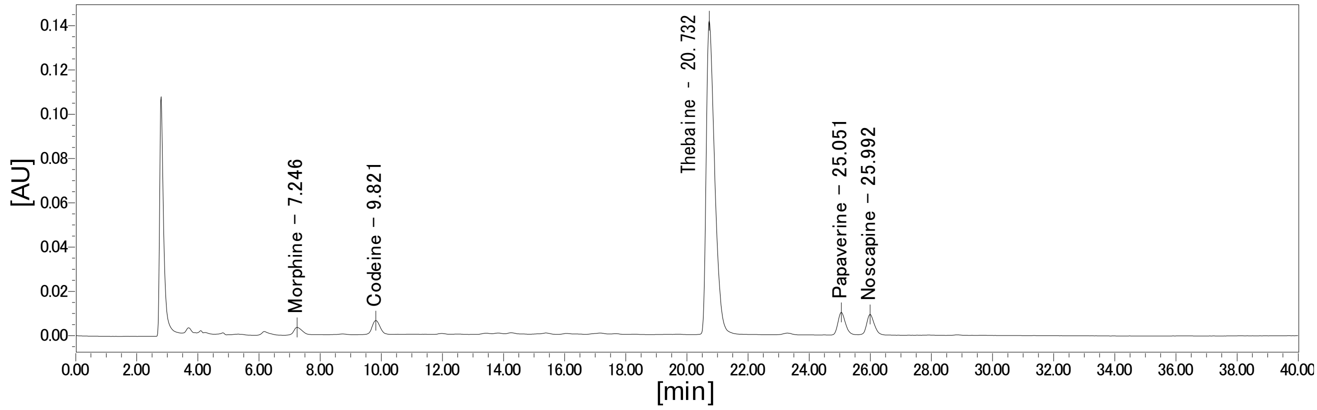

(**C**) T_1 #1-27(HT)

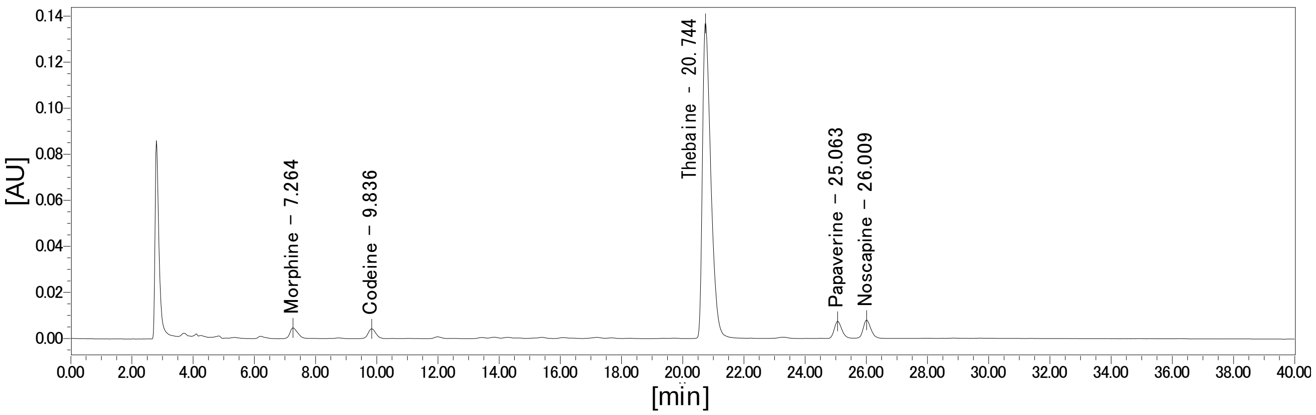

(**D**) T_1 #2-17(HT)

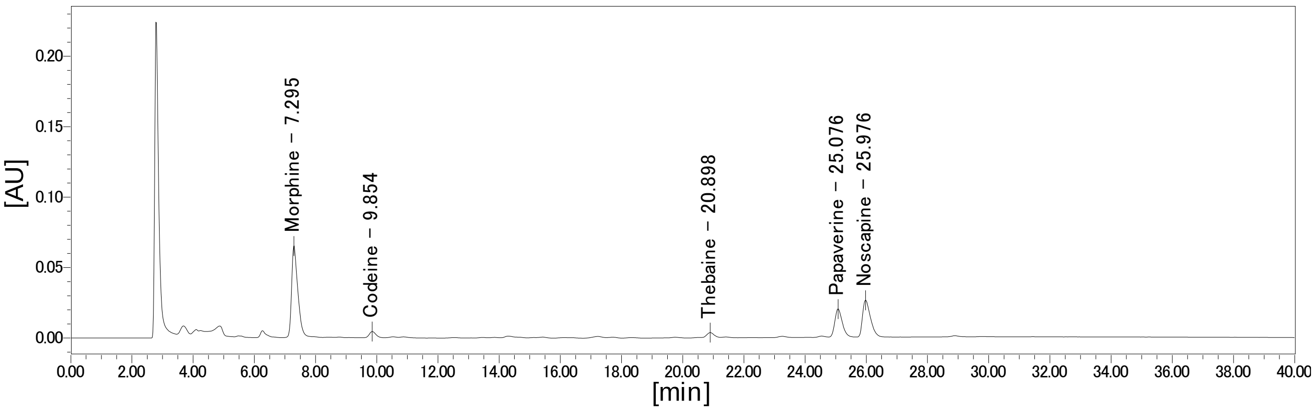

(**E**) T_1 #2-1(LT)

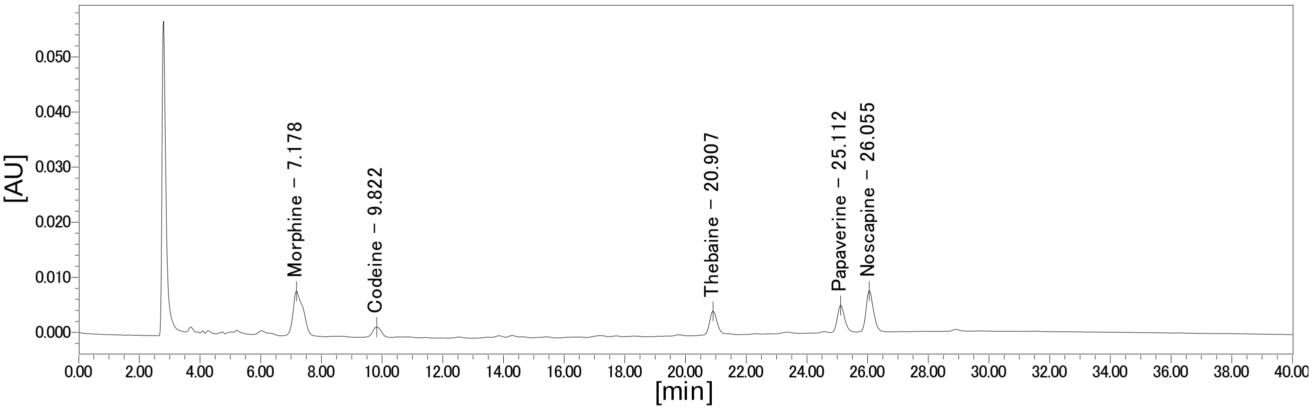

(**F**) T_1 #2-6(LT)

Supplementary Table 1. Summary of the used PCR methods, DNA libraries, primer sets, and obtained amplified DNA fragments in the analyses of T-DNA insertion loci.

PCR Method	Region	Library *	Primer Set				Description of Amplified Product(s)
			1st PCR		2nd PCR		
Inverse PCR	LB	*Hae*III	490S	401A	510S	368A	Tandem 1, 2, 5, 6; riorf13-1, 2
	LB	*Kpn*I	75S	58A	80S	52A	Tandem 2
	LB	*Kpn*I	490S	401A	510S	368A	LB1; Tandem 1
	RB	*Bam*HI	14738S	12909A	14765S	12878A	RB4
	RB	*Eco*RV	14738S	14408A	14765S	14307A	RB1, 2, 3; Tandem 1
	RB	*Eco*RV	14997S	14981A	15011S	14960A	riorf13-3
	RB	*Kpn*I	14997S	14981A	15011S	14960A	Tandem 1

Supplementary Table 1. *Cont.*

PCR Method	Region	Library *	Primer Set				Description of Amplified Product(s)
			1st PCR		2nd PCR		
Inverse PCR	RB	*Pvu*II	14738S	12909A	14765S	12878A	RB4
	RB	*Ssp*I	14738S	14408A	14765S	14307A	RB4; Tandem 1, 4
	RB	*Stu*I	14738S	14408A	14765S	14307A	RB4; Tandem 5
Adaptor-ligation PCR	LB	*Eco*RV	AP1	401A	AP2	368A	Tandem 2; riorf13-1, 2
	LB	*Hae*III	AP1	401A	AP2	368A	LB2, 3, 4; Tandem 5
	LB	*Pvu*II	AP1	401A	AP2	368A	LB2; Tandem 1, 2; riorf13-1, 2
	LB	*Ssp*I	AP1	237A	AP2	226A	LB2; Tandem 1, 2, 5; riorf13-1, 2
	LB	*Stu*I	AP1	401A	AP2	368A	Tandem 4, 6; riorf13-1
	RB	*Eco*RV	14738S	AP1	14765S	AP2	RB1, 2, 3; Tandem 1; riorf13-3
	RB	*Hae*III	14963S	AP1	14997S	AP2	RB5, 6; Tandem 6; riorf13-4
	RB	*Pvu*II	14738S	AP1	14765S	AP2	RB3, 4; riorf13-4
Genome direct PCR	RB, LB	un cut	14738S	401A	14765S	368A	Tandem 1
	RB, LB	un cut	14738S	401A	**		Tandem 1, 3, 4

* Restriction enzymes used for library construction are shown; ** 1st PCR only, anneal 62 ℃.

Supplementary Table 2. List of the sequences (from 5' to 3') of the primers and the oligo-DNAs used for IPCR, AL-PCR, and genome direct PCR.

Left Border Primers			
Sense Primers		Anti-Sense Primers	
75S	ATT ACC AGA TGG TGT CCG GG	52A	TCG CTT CAA AGC TCT TGT AC
80S	CAG ATG GTG TCC GGG TAT G	58A	CGA AAA TCG CTT CAA AGC TC
490S	GAG CAA CGA CGT TTC AGT GC	226A	TTG TTG CAC GGC GAC GAT TGG
510S	ATT CCT GTA CTC GCT TTC CG	237A	AGG CTC ACG TAT TGT TGC ACG
		368A	AAC TCG TAC GGC ATA GTC GG
		401A	TCA ACT TGT GGC GAG TCT CC
**Right Border Primers**			
**Sense Primers**		**Anti-Sense Primers**	
14738S	TAA CCT CGC ACG TAT GGT GC	12878A	CAT GCT CAT GCT TTA GCA GG
14765S	GAC GTC GAT TTC GAT GTG G	12909A	TGC CTT CCA ATT GAC GCT C
14963S	AGC CGA CAA CGT GCA ATT TGC	14307A	CAA TTT TCT TGA CCG AAG CC
14997S	GAG CCA CCT GCC TTC AGA CC	14408A	AGG TGC CAC AGT CCG ACT G
15011S	CAG ACC TAC TGT TTG CTG CG	14960A	ATA ATT TAG AGC ATT GCG GG
		14981A	AAA TTG CAC GTT GTC GGC TG

Adaptor Oligo DNAs	
AP-LS	CGTTGAGAGGCATTATCTTCGGGCTAAGGCGTCGTCGCGACGTCACGC
AP-SS *	(PO_4)-GCGTGACGTC-(NH_2)
**Adaptor Specific Primers**	
AP1	CGT TGA GAG GCA TTA TCT TCG G
AP2	TTA TCT TCG GGC TAA GGC G

* AP-SS: 5'-end phosphorylated, 3'-end amino-linked.

Supplementary Table 3. List of the primers used for the analysis of inheritance manner of T-DNA integration loci by PCR.

Target Region	Primer Name	Orientation	Sequence (from 5' to 3')
Left border genomic region primers			
LB1g	LB1gex-S1	sense	TAA TCA CGA TGT ACC CGT AGC
LB2g	LB2g-1072S	sense	CGT CGT TGT ATG ATG AAT CGC
LB3g	HaeLB3g-S1	sense	CAA CAG TTC GCA GGC AAG TC
LB4g	HaeLB4g-S1	sense	TGA CTA ATC CTG ACT CCA ACC
Right border genomic region priers			
RB1g	RB1g-460A	anti-sense	AGA ACC TTC GTA TGG CAG ATG
RB2g	RB2g-393A	anti-sense	CAA TCC TGC CTC CTA CAA TTC
RB3g	RB3g-588A	anti-sense	GGT CCA TTG ATA TGG CTA CTC
RB4g	RB4g-261A	anti-sense	TCG AAT CGA CGT AAA CTC TCC
RB5g	HaeRB5g-A1	anti-sense	CCT GCA ACA ACT AAC AAC GAG
RB6g	HaeRB6g-A1	anti-sense	GGG TGG TAT TGT AAC TTC ACT C
T-DNA border primers			
T-DNA RB	MAFF-226A	anti-sense	TTG TTG CAC GGC GAC GAT TGG
T-DNA LB	MAFF-14963S	sense	AGC CGA CAA CGT GCA ATT TGC

Supplementary Table 4. List of the primers used for the estimation of T-DNA copy number by real-time PCR.

Target Region	Primer Name	Orientation	Sequence (from 5' to 3')
LB1-genome DNA (LB1g)	LBm-S	sense	GCAGTAGTATCACTGACAAATCGCAC
	LBm-A	anti-sense	CCAGTAACACCCTGGGAATCC
LB1-junction (LB1j)	LBjm-S	sense	GGGGTAAACTCATCCAAACACAC
	LBjm-A	anti-sense	AAAACGTGCTCGAAAATCGC
orf2	orf2m-S	sense	TCCGATGGAGCATTTCTATGG
	orf2m-A	anti-sense	ATGATCTACACGCGGAAGAAGC

Supplementary Table 5. List of the primers used for the RT-PCR analysis of selected morphine biosynthetic genes.

Target Gene	Accession No.	Primer Name	Orientation	Sequence (from 5' to 3')
CYP80B3	AF134590	PsCYP80B3-878S	sense	CAATAGAATGGGCGTTGTCTG
		PsCYP80B3-1377A	anti-sense	ATGATCAGTTGGCTAGGATCC
4'OMT1	AY217333	Ps4'OMT-552S	sense	AGATTGTAGAGATACTTTTCAAGG
		Ps4'OMT-1021A	anti-sense	TGATCTTATGAGACCGAAAGC
SalAT	AF339913	PsSalAT-832S	sense	TCTCTAATATGGAAGTCCGTG
		PsSalAT-1389A	anti-sense	GAACTTAGCCATATCATCCTC
Cor1-1	AF108432	PsCor1-467S	sense	TTGGCTTCACTAGGGCAATCG
		PsCor1-958A	anti-sense	CTCATCCCAGAACTCTTCTTC
Cor2-1	AF108438	PsCor2-463S	sense	ATGCTTGGATTAACCAAGTCG
		PsCor2-887A	anti-sense	AAGGATCACCGGTTGACACTC
T6ODM	GQ500139	T6ODM-S2	sense	CTCTACAAGTACAAGCAGCC
		T6ODM-RTa	anti-sense	TCTGACATGCAATCACGTCG
CODM	GQ500141	CODM-RTs	sense	GACTTATTTGAAGATGGGTTG
		CODM-A2	anti-sense	CTTCAAAATATCCTCATACCTACC
PsACT1	AB574417	PsACT1-145S	sense	CACACTGTGCCAATCTATGAG
		PsACT1-488A	anti-sense	TGGATACCAGCAGCTTCCATC
